# EFFECTS OF A 6-WEEK TASK SPECIFIC POWER TRAINING WITH AND WITHOUT COGNITIVE TRAINING AMONG OLDER ADULTS WITH SLOW GAIT

**DOI:** 10.1093/geroni/igz038.3208

**Published:** 2019-11-08

**Authors:** Elisa F Ogawa, Rebekah Harris, Joseph DeGutis, Rachel Ward, Jennifer Brach, Ildiko Halasz, Jonathan Bean

**Affiliations:** 1 New England GRECC, VA Boston Healthcare System, Boston, Massachusetts, United States; 2 NE GRECC, VA Boston Healthcare System, Boston, Massachusetts, United States; 3 Department of Psychiatry, Boston, Massachusetts, United States; 4 School of Health and Rehabilitation Sciences, University of Pittsburgh, Pittsburgh, Pennsylvania, United States; 5 Department of Medicine, VA Boston Healthcare System, Boston, Massachusetts, United States

## Abstract

Task-specific power training (InVEST) targets leg power and mobility skills that are beneficial for treating slow gait speed for older adults. This study investigated the efficacy of a short-term InVEST training on leg power, mobility performance, and gait characteristics and further examine whether the addition of cognitive training would augment the impact on the outcomes. Mobility limited community-dwelling older Veterans age ≥65 years were recruited. Participants were randomly assigned to either InVEST training (n=10) or InVEST+cognitive training (n=11). Training occurred 3 times per week for 6 weeks. Sessions were either 70 minutes (InVest+cognitive training) or 40 minutes (InVEST) in duration. Leg power, mobility performance (Short Physical Performance Battery), and gait characteristics (gait speed, stance time, step width, swing time, step length and their variabilities under single-task, simple and complex dual-task walking conditions) were evaluated. Twenty-one men with mean age 76±7 years completed the study and 86% were of white race. Among all participants, clinically relevant and statistically significant improvements in leg power, mobility performance, and gait characteristics (gait speed, step length, stance time under all three gait conditions) were observed. There were no statistically significant or clinically relevant group differences among any of the outcomes based on cognitive training status. Short-term InVEST training led to clinically meaningful improvements in leg power, mobility performance, and gait characteristics. These findings add to the body of evidence supporting the benefits of InVEST training on mobility and do not support the contention that mixed modes of training (cognitive and physical) may augment mobility outcomes.

